# Treatment outcome of extra-pulmonary tuberculosis in Finland: a cohort study

**DOI:** 10.1186/1471-2458-10-399

**Published:** 2010-07-06

**Authors:** Tuula Vasankari, Pekka Holmström, Jukka Ollgren, Kari Liippo, Petri Ruutu

**Affiliations:** 1 Department of Infectious Disease Surveillance and Control, National Institute for Health and Welfare, Mannerheimintie 166, 00300 Helsinki, Finland; 2 Department of Respiratory Medicine, Turku University Hospital, Paimio Hospital, Alvar Aallon tie 275, 21540 Preitilä, Finland

## Abstract

**Background:**

We investigated the treatments given, the outcome and the patient- and treatment-system dependent factors affecting treatment outcome in a national two-year cohort of culture-verified extra-pulmonary tuberculosis cases in Finland.

**Methods:**

Medical records of all cases in 1995 - 1996 were abstracted to assess treatment and outcome, using the European recommendations for outcome monitoring. For risk factor analysis, outcome was divided into three groups: favourable, death and other unfavourable. Predictors of unfavourable outcome were assessed in univariate and multivariate analysis.

**Results:**

In the study cohort of 276 cases, 116 (42.0%) were men and 160 (58.0%) women. The mean age was 65.7 years. A favourable outcome was achieved in 157/276 (56.9%) cases, consisting of those cured (8.0%) and treatment completed (48.9%). Death was the outcome in 17.4% (48/276) cases, including cases not treated. Other unfavourable outcomes took place in 45 (16.3%) cases. Significant independent risk factors for death in multinomial logistic regression model were male sex, high age, immunosuppression, any other than a pulmonary specialty being responsible at the end of the treatment and other than standard combination of treatment. For other unfavourable treatment outcomes, significant risk factor was treatment with INH + RIF + EMB/SM. Deep site of TB was inversely associated with the risk of other unfavourable outcome.

**Conclusions:**

The proportion of favourable outcome was far below the goal set by the WHO. Age and comorbidities, playing an important role in treatment success, are not available in routine outcome data. Therefore, comparisons between countries should be made in cohort analyses incorporating data on comorbidities.

## Background

World Health Organization (WHO) has set the international target value for the favourable treatment outcome to be 85% [1]. WHO and the International Union Against Tuberculosis and Lung Disease (IUATLD) have published joint recommendations for assessing the outcome of tuberculosis treatment aiming at standardised reporting in Europe [2-5]. These recommendations are mainly designed for smear positive pulmonary tuberculosis treated with standard short course protocol, but they are commonly used for extra-pulmonary tuberculosis [6,7].

Many industrialized countries with comprehensive health care and a secure supply of drugs free of charge for patients have not reached the overall objective of 85% with successful outcome, set by WHO [6-17]. In the routine data collection of the European Union and Western European countries among previously untreated definite pulmonary TB cases in 2005, 79% had a successful outcome, 6% died, 4% failed or continued treatment beyond 12 months and 10% were lost to follow up. In our recent cohort analysis of treatment outcome of culture-verified pulmonary tuberculosis the proportion with favourable outcome was only 65%, in contrast to the overall WHO target of 85% [18].

There is limited information concerning countrywide treatment outcome and risk factors for unfavourable treatment outcome of extra-pulmonary tuberculosis in low incidence countries. A problem in assessing treatment outcome in extra-pulmonary tuberculosis is the diversity in the nature of disease depending on the site of disease. In Western Europe, successful outcome was reported for 81% in extra-pulmonary tuberculosis [19]. Most studies including extra-pulmonary cases have reported results combined with pulmonary cases [7,10,20]. In a Danish national cohort analysis, the proportion with favourable outcome in extra-pulmonary tuberculosis was 68% [6].

Our aim was to find out how extra-pulmonary tuberculosis is treated, as well as determine the treatment outcome in Finland. We analyzed the risk factors, including the patient and treatment system dependent factors, for unfavorable treatment outcome of extra-pulmonary tuberculosisin a national, population-based two-year cohort of all culture-verified extra-pulmonary tuberculosis cases to establish a basis for improving treatment outcome results.

## Methods

### Study cohort, case definitions and data collection

The method of identifying all culture-confirmed tuberculosis cases in Finland, with the first positive culture sample date between January 1^st^, 1995, to December 31^st^, 1996 (N = 1059), present in either the National Infectious Disease Register (NIDR) or acquired through a separate query to all microbiological laboratories has been described elsewhere [21].

A case of non-pulmonary tuberculosis was defined as culture finding for *M. tuberculosis *in other specimen type than respiratory secretion, in the absence of *M. tuberculosis *in culture from respiratory secretions and sputum smear positivity for acid fast bacilli [18,22]. With this definition, 322 (30% of the whole cohort) constituted the base-frame for the extra-pulmonary tuberculosis cohort.

**Figure 1:**
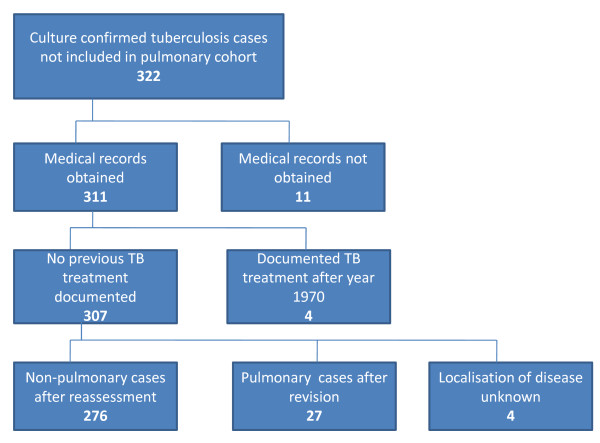
**Composition of the national study cohort of culture-confirmed extra-pulmonary tuberculosis cases**.

Out of the 322 cases in the extra-pulmonary tuberculosis cohort, complete medical records were available for 311 (96.6%) (Figure 1). Among these 311 cases, four (1.3%) had previously been treated for tuberculosis after the year 1970, and were excluded from the outcome analysis as re-treatment cases. We further assessed the detailed clinical information in patient charts in order to divide the cases as deep and superficial disease. During this assessment we identified sample types not directly classifiable as pulmonary or non-pulmonary. All the patient charts and autopsy reports of this group of cases were assessed. We found 27 cases as having been erroneously classified by our original algorithm as extra-pulmonary TB. These cases were diagnosed from the culture of gastric aspirate or biopsy samples from lung tissue. In four cases the information was not sufficient for judging the location of the disease. These 31 cases were excluded from the extra-pulmonary study group leaving 276 extra-pulmonary cases with no previous treatment documented (Table 1). These extra-pulmonary TB cases were divided to deep and superficial disease based on the site of bacteriological sampling and/or ICD-codes. Lymph node and skin tuberculosis were considered as superficial and other forms as deep extra-pulmonary disease. If the same patient had both deep and superficial disease, the case was classified as deep disease.

**Table 1:** The composition of extra-pulmonary study cohort

Form of tuberculosis	N	Percent
Lymp node	106	38.4

Skin	13	4.7

Pleural	50	18.1

Uninary and reproductive system	60	21.7

Bone and joints	20	7.3

Other	27	9.8

Total	276	100.0

### Definitions of treatment

Tuberculosis treatment was initiated in and supervised by the pulmonary departments of public hospitals in the great majority of cases. Chemotherapy combination was decided by the treating physician. In our retrospective record review, the combination actually given to each patient was grouped into six categories (Table 2) in order to describe the variability in treatment combinations used. Definitions for the grouping were based on the national recommendations in Finland following the recommendations by WHO, ATS and BTS [23-28]. We have previously described the treatment grouping in detail [18]. Pauses of chemotherapy were recorded only when lasting at least one week, and calculated only for standard treatments.

**Table 2:** Definitions used in describing the treatment given to the cases in a national cohort of patients with extra-pulmonary tuberculosis

Treatment group	Drugs used in intensive phase	Duration of intensive phase [days]	Drugs used in continuation phase	Total duration of treatment [months]
Standard treatment A	isoniazid + rifampicin + pyrazinamide	At least 54	isoniazid + rifampicin	At least 5 1/2

Standard treatment B	isoniazid + rifampicin + ethambutol or streptomycin	At least 54	isoniazid + rifampicin	At least 8

Standard treatment with short intensive phase C	isoniazid + rifampicin + pyrazinamide or ethambutol or streptomycin	Less than 54	isoniazid + rifampicin	At least 5 1/2 for A At least 8 for B

Standard treatment D	>4 tuberculosis drugs, including the drugs in Standard treatment A or B	At least 54	isoniazid + rifampicin + any other anti-tuberculosis drug(s)	At least 5 1/2 for A At least 8 for B

Other combination of tuberculosis drugs	Non-standard combinations of tuberculosis drugs, excluding the combinations above	NA^2^	Any combination of antituberculosis drugs	NA

Ineffective treatment	One antituberculosis drug used alone or in combination with a drug with limited antituberculosis activity ^1^	NA	NA	NA

^1^E.g. fluoroquinolones

^2^NA = not applicable

### Definitions of outcome

The categories of WHO/EuroTB recommendation for outcome monitoring are cure, treatment completed, failure, death, treatment interrupted (default), transfer out, and on treatment at 12 months [5]. The duration of the follow up period is defined as 12 months from the beginning of the treatment or the date of diagnosis, and the first outcome registered as final.

The WHO/EuroTB category 'treatment interrupted' includes all interruptions, whether caused by a patient or by a treating physician. For analysing these two separately, we divided 'treatment interrupted' into 'physician's decision to stop early' and 'default' for interruptions due to patient only [18]. The outcome was recorded as 'death', if the case died before starting the treatment, during the treatment, or the date of death was within 14 days after cessation of the anti-TB drugs.

The outcome categories used are listed in Table 3. The outcome was categorized as favourable in cases of cure and treatment completed, and as unfavourable in cases of failure, death, default, physician's decision to stop early and transfer out.

**Table 3:** Summary of the outcome criteria used in the study

Indicator	Definition
Cure	Treatment completion and: - culture becoming negative on samples taken at the end of treatment and on at least one previous occasion or - sputum microscopy becoming negative for AFB at the end of treatment and on at least one previous occasion.

Treatment completed	Treatment completion, not meeting the criteria to be classified as Cured or Failed

Death	Death before starting treatment or during treatment, irrespective of cause.

Transfer out	Patient referred to another clinical unit for treatment and final outcome unknown

Failed	Culture or sputum microscopy remaining positive or becoming positive again at 5 months or later during treatment.

Default	Treatment interrupted for 2 consecutive months, or more for reasons due to patient.

Physician's decision to stop early	Treatment interrupted for 2 consecutive months, or more due to physician's decision.

On treatment at 12 months	Patient still on treatment at 12 months who did not meet any other outcome during treatment. This includes patients with: - initial treatment changed due to polyresistance (ie. resistance to at least two first line drugs) on the isolate taken at the start of treatment. - treatment prolonged because of side effects/complications - initial regimen planned for >12 months - information on the reasons for being still on treatment not available

Not known	Information on outcome not available, for cases not known to have been transferred

### Definitions of origin, social and medical risk factors

A case was defined as immigrant if the country of birth was not Finland or, in the absence of country of birth, the nationality was other than Finnish. Immunosuppressive treatment was defined as corticosteroid treatment (>40 mg per day of any duration, or any daily dose with treatment duration exceeding one month), cytotoxic or cyclosporine treatment, or radiation therapy during the preceding year. For inclusion in the group of social risk factors, a case had a history of alcohol abuse, unemployment, imprisonment or homelessness recorded in patient records. To be included in the diabetes risk group the case had juvenile or adult onset disease on medication.

### Definitions of characteristics of treatment system

Specialty responsible for treatment was that of the unit treating patient as an inpatient or outpatient.

### Ethical review

The ethics approval for this study was acquired from the National Research and Development Centre for Welfare and Health.

### Statistical methods

Median age comparisons where tested using Median test. For comparison of proportions, Chi-square or Fisher's generalised exact test with Monte Carlo simulation was used. Stata 11.0 (StataCorp LP Texas, USA) and IBM SPSS for Windows, version 18.0.2 (Chicago, IL, USA) was used for statistical calculations. We used binary and multinomial logistic regression model to assess the relationships between all predictors that were found to be significant in pulmonary tuberculosis (to compare extra-pulmonary tuberculosis to pulmonary tuberculosis in respect to these variables), and other variables which in the univariate analysis had a p-value less than 0.20, with binomial and trinomial outcome variables, in which the reference class was favourable outcome. To the final model, predictors were selected by forcing all those variables that were significant in pulmonary tuberculosis, and from other variables selection was by forward stepwise method. P-values under 5% were considered as significant. For univariate results also Chi squared test and Fisher's exact test were used.

## Results

In the study cohort of 276 cases, 116 (42.0%) were men and 160 (58.0%) women. The median age was 70.1 years. There were 170 cases aged >65 years (61.6%). The proportion of immigrants was 10.9% (30 cases), mainly from developing countries. There were no cases of HIV-coinfection in the cohort. There was one prisoner in the study cohort.

### Treatment given

Tuberculosis chemotherapy was given to 257 (93.1%) of the 276 cases. Half of the cases (140/257; 54.5%) received standard treatment A, 28 (10.9%) received standard treatment B, and 30 (11.7%) received standard treatment with additional drugs (D) (Table 4). In 18 (7.0%) of the cases the intensive phase was short (category C). A total of 41 cases (16.0%) received either other combinations of tuberculosis drugs or ineffective treatment. Most of the cases (177/257, 68.9%), were treated with the same combination during the whole treatment. Nineteen (6.9%) of the 276 cases were untreated, the majority (16; 84.2%) of whom died before the diagnosis of tuberculosis was established.

**Table 4:** Treatment outcome in the study cohort of 276 extra-pulmonary tuberculosis cases according to the WHO/EuroTB classification, modified for 'default' and 'physician's decision to stop early'

Treatment group	FAVOURABLE			UNFAVOURABLE¹					On treatment at 12 months	Not known	Total
					
	Cured	Treatment completed	Subtotal	Death	Transfer out	Default	Physician's decision to stop early	Subtotal			
Standard treatment A	15 10.7%	82 58.6%	97 69.3%	18 12.9%	0 0	3 2.1%	14 10.0%	35 25.0%	7 5.0%	1 0.7%	140 100%

Standard treatment B	1 3.6%	9 32.1%	10 35.7%	5 17.9%	0	1 3.6%	8 28.6%	14 50.0%	4 14.3%	0	28 100%

Standard treatment with short intensive phase C	0	14 77.8%	14 77.8%	1 5.6%	0	0	2 11.1%	3 16.7%	1 5.6%	0	18 100%

Standard treatment D	3 10.0%	10 33.3%	13 43.3%	1 3.3%	0	1 3.3%	4 13.3%	6 20.0%	11 36.7%	0	30 100%

Other combination of tuberculosis drugs	3 8.3%	16 44.4%	19 52.7%	6 16.7%	1 2.8%	5 13.9%	3 8.3%	15 41.7%	1 2.8%	1 2.8%	36 100%

Ineffective treatment	0	4 80.0%	4 80.0%	1 20.0%	0	0	0	1 20.0%	0	0	5 100%

Overall outcome with any treatment	22	135 52.5%	157 61.1%	32 12.5%	1 0.4%	10 3.9%	31 12.1%	74 28.8%	24 9.3%	2 0.8%	257 100%

No treatment	NA	NA	NA	16 84.2%	0	3² 15.8%	0	19 100%	0	0	19 100%

Total	*22* *8.0%*	*135* *48.9%*	*157* *56.9%*	*48* *17.4%*	*1* *0.4%*	*13* *4.7%*	*31* *11.2%*	*93* *33.7%*	*24* *8.7%*	*2* *0.7%*	*276* *100%*

¹There were no failures in the cohort.

²The treating physician decided not to give any treatment.

The median age differed significantly between the treatment groups; it was 70,5 (range 6,9-94,4) years for category A, 72.6 (18,5-90,4) for category B, 58.2 (6,6-91,0) for category C, 57.5 (15,1-88,2) for category D, 72.6 (60,8-71,6) for other combinations and 67.8 (18,6-94,4) for ineffective treatment (p = 0.005). There were 62 (44.3%) men in treatment group A, 10 (35.7%) in B, 6 (33.3%) in C, 15 (50.0%) in D, 8 (22.2%) in other combinations and 4 (80.0%) in ineffective treatment group. The difference was significant (p = 0.04). There was no statistical difference between groups regarding the presence of a social risk factor (p = 0.32).

### Outcome of treatment

A favourable outcome was achieved in 157/276 (56.9%) of the cases, consisting of those cured (8.0%) and treatment completed (48.9%). The proportion with favourable outcome varied between 35.7 to 80.0% in different treatment groups. There were no treatment failures in the cohort.

The proportion of cases defaulting or transferring out was 14/276 (5.1%), and of them 5/14 (35.7%) were immigrants and 2/14 (14.3%) had a social risk factor. In 31 (11.2%) cases treatment was stopped prematurely by physician. Death was the outcome in 17.4% (48/276) cases, including cases not treated.

The proportion of cases with favourable outcome differed significantly between treatment groups (P < 0.001), and was smallest in treatment groups B and D (35.7% and 43.3%). The proportion of deaths differed between groups, and was lowest in treatment group D (3.3%). In the ineffective treatment group the proportion of deaths was higher (20.0% versus 12.1%) than in the other combinations of TB drugs.

24 of the cases were still on treatment at 12 months, and in two cases it was not possible to judge the outcome from the information available. Of the remaining 250 cases, 19 were not treated, among them 11 (57.9%) were men and 8 (42.1%) women. Of this group, with a median age of 81.0 years, 16 died before treatment and 3 were left untreated. These 45 cases were excluded from the analysis of risk factors of poor treatment outcome.

### Analysis of risk factors for unfavourable outcome

In univariate analysis, patient-related risk factors which were significantly associated (p < 0.05) with death, were high age, immunosuppression and diabetes (Table 5). Treatment system -related risk factors which were significantly associated with death were the specialty of the treating unit (internal medicine, general medicine in primary care), change of specialty responsible for treatment and less than five treated cases per year per unit (Table 6). There was no statistically significant patient-related personal risk factor association for other unfavourable outcome (i.e. transfer out, default or physician's decision to stop early) (Table 5). Treatment system -related significant risk factors for other unfavourable outcome were standard treatment B, other treatment combination, change in treatment group and pause(s) during treatment (Table 6). For all unfavourable outcomes together, i.e. death and other unfavourable outcome combined, significant predictors in univariate analysis were high age, immunosuppression, specialty of the treating unit (general medicine in primary care), change of specialty responsible for treatment, less than five treated cases per year per unit, standard treatment B, other treatment combination and pause(s) during treatment.

**Table 5:** Univariate analysis of the association of patient -related characteristics with an unfavourable outcome in 231 cases treated for at least 24 hours

Variable		Total	Death			Other unfavourable			All unfavourable (death and other) together		
		
		N	N	OR (95% CI)	p	N	OR (95% CI)	p	N	OR (95% CI)	p
Sex	female	137	15	1		26	1		41	1	
	
	male	94	17	1.78 (0.83-3.82)	0.14	16	0.97 (0.48-1.95)	0.93	33	1.27 (0.72-2.21)	0.41

Age at diagnosis	risk per five years			1.53 (1.24-1.88)	**<0.001**		1.07 (0.95-1.21)	0.29		1.18 (1.03-1.34)	**0.01**

TB history	no	200	30	1		35	1		65	1	
	
	yes	31	2	0.41 (0.09-1.83)	0.24	7	1.23 (0.49-3.11)	0.67	9	0.85 (0.37-1.95)	0.70

Social risk factor	no	215	31	1		40	1		71	1	
	
	yes	16	1	0.36 (0.05-2.83)	0.33	2	0.55 (0.12-2.56)	0.45	3	0.47 (0.13-1.67)	0.25

Site of tuberculosis	superficial	114	13	1		26	1		39	1	
	
	deep	117	19	1.34 (0.62- 2.89)	0.46	16	0.56 (0.28-1.13)	0.11	35	0.82 (0.47-1.43)	0.48

Immunosuppression	no	204	22	1		38	1		60	1	
	
	yes	27	10	5.03 (2.00-12.87)	**0.001**	4	1.17 (0.36-3.78)	0.80	14	2.58 (1.15-5.83)	**0.02**

Malignancy¹	no	223	29	1		42	1		71	1	
	
	yes	7	2	2.02 (0.38-10.9)	0.41	0	0	1.00	2	0.84 (0.16-4.46)	0.84

Diabetes	no	205	25	1		37	1		62	1	
	
	yes	26	7	2.86 (1.05-7.79)	**0.04**	5	1.38 (0.47-4.08)	0.56	12	1.98 (0.86-4.52)	0.11

Total		231	32			42			74		

¹ Information is missing for one case.

² Max likelihood estimate is not finite.

Significant p-values are darkened.

**Table 6:** Univariate analysis of the association of treatment system -related characteristics with an unfavourable outcome in 231 cases treated for at least 24 hours

Variable		Total	Death			Other unfavourable			All unfavourable (death and other) together		
		**N**	**N**	**OR (95% CI)**	**p**	**N**	**OR (95% CI)**	**p**	**N**	**OR (95% CI)**	**p**

Specialty responsible for starting treatment	pulmonary	185	18	1		34	1		52	1	
	
	internal medicine	33	9	3.50 (1.38-8.90)	**0.009**	5	1.02 (0.36-2.96)	0.96	14	0.43 (0.06-2.97)	0.39
	
	general medicine	2	2	Inf³	1.00	0	1.05	1.00	2	0.23 (0.04-1.20)	0.08
	
	other	11	3	4.43 (0.98-20.14)	0.05	3	2.35 (0.53-10.31)	0.26	6	0.61 (0.12-2.10)	0.37

Specialty responsible for ending treatment	pulmonary	161	8	1		34	1		42	1	
	
	internal medicine	29	7	6.13 (1.97-19.05)	**0.002**	5	1.03 (0.35-2.99)	0.96	12	2.00 (0.88-4.53)	0.10
	
	general medicine	35	16	14.00 (5.21-37.65)	**<0.001**	2	0.41 (0.09-1.87)	0.25	18	3.00 (1.42-6.35)	**0.004**
	
	other	6	1	3.72 (0.37-37.29)	0.26	1	0.41 (0.09-8.09)	0.91	2	1.42 (0.25-8.02)	0.69

Change of specialty responsible for treatment	no	174	16	1		32	1		48	1	
	
	yes	57	16	4.06 (1.83-9.02)	**0.001**	10	1.27 (0.56-2.86)	0.58	26	2.20 (1.19-4.09)	**0.01**

Number of cases per year for unit responsible for ending treatment	1 - 4	55	16	4.37 (1.86-10.28)	**0.001**	10	1.49 (0.63-3.50)	0.36	26	2.51 (1.30-4.84)	**0.006**
	
	5 - 10	12	1	0.88 (0.10-7.56)	0.91	2	0.96 (0.19-4.76)	0.96	3	0.93 (0.24-3.64)	0.92
	
	11 - 29	34	3	1.03 (0.27-3.96)	0.96	8	1.50 (0.59-3.80)	0.39	11	1.34 (0.59-3.03)	0.49
	
	30 -	129	12	1		22	1		34	1	

Treatment group	standard treatment A	132	18	1		17	1		35	1	
	
	standard treatment B	24	5	2.69 (0.82-8.82)	0.10	9	5.14 (1.82-14.49)	**0.002**	14	3.88 (1.58-9.53)	**0.003**
	
	standard treatment C	17	1	0.40 (0.05-3.11)	0.37	2	0.82 (0.17-3.91)	0.80	3	0.59 (0.16-2.19)	0.43
	
	standard treatment D	19	1	0.41 (0.05-3.37)	0.41	5	2.19 (0.69-6.95)	0.18	6	1.28 (0.45-3.62)	0.64
	
	other combination	34	6	1.70 (0.60-4.84)	0.32	9	2.70 (1.05-6.96)	**0.04**	15	2.19 (1.00-4.77)	**0.049**
	
	ineffective	5	1	1.35 (0.14-12.76)	0.80	0	0	1.00	1	0.69 (0.07-6.41)	0.74

Change in treatment group	no	159	22	1		22	1		44	1	
	
	yes	72	10	1.00 (0.43-2.34)	0.99	20	2.12 (1.05-4.27)	**0.04**	30	1.56 (0.87-2.80)	0.13

Pause of treatment	no	169	25	1		22	1		47	1	
	
	yes	62	7	0.98 (0.39-2.45)	0.96	20	3.17 (1.55-6.46)	**0.002**	27	2.00 (1.09-3.66)	**0.02**

Pause during intensive phase¹	no	183	25	1		24	1		49	1	

	yes	47	6	1.40 (0.52-3.78)	0.51	18	4.37 (2.05-9.29)	**<0.001**	24	2.88 (1.43-5.78)	**0.003**

Pause during intensive phase, due to side effect	no	191	26	1		27	1		53	1	
	
	yes	40	6	1.68 (0.61-4.60)	0.32	15	4.04 (1.82-8.92)	**0.001**	21	2.85 (1.48-5.51)	**0.002**

Pause during continuation phase	no	171	12	1		25	1		37	1	
	
	yes	34	2	1.12 (0.23-5.36)	0.89	12	3.22 (1.40-7.40)	**0.006**	14	2.53 (1.17-5.50)	**0.02**
	
	NA (other + ineffective)	26	18			5			23		

Total		231	32			42			74		

Significant p-values are darkened.

¹ Information missing for one case

² Information missing for 3 cases

³ Max likelihood estimate is not finite.

Significant independent risk factors for death in multinomial logistic regression model were male sex, high age, immunosuppression, any other than a pulmonary specialty being responsible at the end of the treatment and other than standard combination of treatment. For other unfavourable treatment outcomes, significant risk factor was treatment group B (Table 7). Deep site of TB was inversely associated with the risk of other unfavourable outcome (p = 0.02).

**Table 7:** Multivariate analysis of 231 cases treated at least 24 hours, odds ratio for death or other unfavourable (transfer out, default, physician's decision to stop early) outcomes.

Variable		Death			Other unfavourable			All unfavourable (death and other) together		
		
		N	OR (95% CI)	p	N	OR (95% CI)	p	N	OR (95% CI)	p
Sex	female	15	1		26	1		41	1	
	
	male	17	8.53 (3.23-22.50)	**<0.001**	16	1.24 (0.55-2.84)	0.61	33	2.04 (0.98-4.24)	0.06

Age at diagnosis	risk per five years		1.10 (1.06-1.14)	**<0.001**		1.01 (0.98-1.04)	0.30		1.16 (1.03-1.31)	**0.02**

Immunosuppression	no	22	1		38	1		60	1	
	
	yes	10	5.58 (1.92-16.21)	**0.002**	4	1.22 (0.38-3.90)	0.74	14	2.07 (1.33-3.22)	**0.001**

TB history	no	30	1		35	1		75	1	
	
	yes	2	0.27 (0.05-1.59)	0.15	7	1.04 (0.44-2.43)	0.93	9	0.74 (0.32-1.70)	0.48

Site of tuberculosis	superficial	13	1		26	1		39	1	
	
	deep	19	1.48 (0.68-3.20)	0.32	16	0.46 (0.24-0.86)	**0.02**	35	0.62 (0.38-1.03)	0.07

Specialty responsible for ending treatment	pulmonary	8	1		34	1		42	1	
	
	internal medicine	7	6.85 (2.24-20.97)	**0.001**	5	1.09 (0.32-3.72)	0.90	12	1.89 (0.86-4.14)	0.11
	
	general medicine	16	28.91 (5.95-140.51)	**<0.001**	2	0.33 (0.08-1.38)	0.13	18	2.59 (0.97-6.91)	0.06
	
	other	1	173.65 (12.97-2325.22)	**<0.001**	1	1.99 (0.79-5.01)	0.14	2	5.27 (1.48-18.73)	**0.01**

Pause of treatment	no	25	1		22	1		47	1	
	
	yes	7	0.25 (0.58-1.07)	0.06	20	2.48 (0.92-6.69)	0.07	27	1.28 (0.54-3.07)	0.58

Treatment group	standard treatment A	18	1		17	1		35	1	
	
	standard treatment B	5	3.88 (0.89-16.87)	0.07	9	3.67 (1.52-8.85)	**0.004**	14	4.32 (2.00-9.31)	**<0.001**
	
	standard treatment C	1	1.49 (0.27-8.12)	0.65	2	0.75 (0.14-4.00)	0.73	3	0.98 (0.22-4.27)	0.98
	
	standard treatment D	1	1.26 (0.10-16.51)	0.86	5	2.52 (0.50-12.80)	0.27	6	2.46 (0.76-8.00)	0.13
	
	other combination	6	5.06 (1.13-22.57)	**0.03**	9	1.47 (0.35-6.24)	0.60	15	2.02 (0.56-7.31)	0.28
	
	ineffective	1	3.44 (0.54-22.00)	0.19	0	0	**<0.001**	1	0.59 (0.11-3.20)	0.54

Significant p-values are darkened.

Reference category is favourable treatment outcome.

For death and other unfavourable outcomes together, significant risk factors in the binary logistic regression model were high age (p = 0.02), immunosuppression (p = 0.001), treatment group B (p < 0.001) and any other than a pulmonary, internal and general medicine specialty being responsible at the end of the treatment (p = 0.01). Almost significant risk factors were male sex (p = 0.06) and specialty of the treating unit (general medicine in primary care) (p = 0.06). Deep site of tuberculosis was almost significantly protective (p = 0.07) (Table 7).

When we analysed all 250 cases in the cohort with known outcome, including those 19 without treatment, the associations observed in multivariate analysis as significant were the same as with the 231 cases of the presented analysis.

## Discussion

In a two-year national cohort of culture-confirmed extra-pulmonary tuberculosis cases, we observed a favourable outcome of treatment in 57%, far less than the target level presented by WHO for favourable outcomes. High death rate, physician's decision to stop the treatment too early and treatment still going on at 12 months were the main reasons.

The proportion of favourable outcome in our extra-pulmonary tuberculosis cohort was even smaller than for culture- proven pulmonary tuberculosis during the same time period [18]. Our proportion with favourable outcome was smaller than that in a Danish national cohort analysis, where the proportion of favourable outcome in extra-pulmonary tuberculosis was 68% [6], or the 81% reported for extra-pulmonary tuberculosis in the routine surveillance data collection of the European Union and other Western European countries [19]. In a number of studies combining outcome in pulmonary and extra-pulmonary cases, the proportions with favorable treatment outcomes have ranged between 68 - 82% [7,10,20], and are not comparable to our current figures. The large proportion of lymph node and skin manifestations in extra-pulmonary tuberculosis, for the outcome of which the impact from systemic chemotherapy may be different from pulmonary and deep extra-pulmonary tuberculosis, warrants separate assessment of the outcome of extra-pulmonary tuberculosis.

The outcome analysis recommended by WHO and the International Union Against Tuberculosis and Lung Disease (IUATLD) is mainly developed for smear or culture positive pulmonary disease. It can be used in extra-pulmonary disease, but due to the great diversity in the clinical presentation of extra-pulmonary disease and treatment regimens used, it might need development for this purpose. For example, in TB meningitis, the proposed treatment duration is 12 months, and the case may therefore be categorized in the still on treatment category. There is also difficulty in acquiring culture samples late in the treatment for defining category cure. On the other hand, the proportion of tuberculosis of different extra-pulmonary organ locations is small in developed countries, and it would be impractical to have many different outcome assessment systems for pulmonary and extra-pulmonary disease.

The proportion of those being still on treatment at 12 months was 8.7%, higher than the 3.9% in the pulmonary cohort [18]. The wide clinical diversity of extra-pulmonary TB can explain this partly, because the treatment time in the tuberculosis of central nervous system and bones is usually longer. It is also possible that the treatment of the wide variety of different forms of extra-pulmonary tuberculosis is even more unfamiliar to doctors than treating pulmonary tuberculosis.

There are no earlier studies of risk factors for unfavorable outcome in extra-pulmonary TB. Compared to those studies including all forms of disease, male sex, high age and immunosuppression were risk factors for death as has been previously reported [6,8,29]. Any other than a pulmonary specialty being responsible at the end of the treatment as a risk factor for death was noticed already in our pulmonary cohort [22], but other than standard combination of treatment as a risk factor was noticed only in this study. Even though our data allowed controlling for a range of comorbid states, it is possible that, in a patient population where half of the cases are older than 65 years, there could be more comorbidity in the subgroup that ends up being treated in internal medicine and geriatric services. In the univariate analysis there was a reverse association of death with the number of cases treated per year by the unit in charge of ending the treatment. However, this association did not remain an independent predictor in multivariable analysis, in contrast to our earlier study on pulmonary tuberculosis [22].

The finding that deep localization of TB was inversely associated with the risk of other unfavourable outcomes than death was new. It is probable that the efforts to ensure secure the continuity of treatment depend on the severity of TB disease. Superficial disease may be considered more benign. Our previous finding in pulmonary tuberculosis of the protective effect of history of earlier tuberculosis was not observed in this study population. The site of disease was not significantly associated with the risk of death. It seems that other factors like age and other comorbidities are more important risk factors of death, and the site of disease (deep or superficial) plays a smaller role.

In our previous analysis on pulmonary tuberculosis in the same two-year cohort of culture-verified tuberculosis, a case was defined as pulmonary using the case definition of NIDR, i.e. as a culture finding for *M. tuberculosis *in sputum or bronchoalveolar lavage (BAL), or as a culture finding for *M. tuberculosis *from another sample type in a case with sputum smear positive for acid fast bacilli. Now the analysis of the remaining part of the two-year cohort, revealed a group of 27 pulmonary cases with biopsy samples or gastric aspirate samples. This same bias existed probably in the statistics of NIDR, but it can be considered small compared to the total figures. The NIDR system is highly computerized, and a thorough evaluation of all TB cases is not possible with the resources available. However all cases in those less than 16 years of age are investigated in detail.

Due to the strictly controlled data collection process and high coverage, as reported previously [21], the data is highly representative. Only in 13 cases we could not trace enough patient record data to classify them. TB treatment recommendations and treatment organisation, the age distribution and the case fatality rate have all remained unchanged since the study period in 1995-1996, making the analysis and conclusions valid for the present.

## Conclusions

The proportion of favourable outcome was far below the goal set by the WHO. Age and comorbidities, which play an important role in treatment success, are not usually analyzed for the routine outcome data. Comparisons in outcome between different countries should be made in cohort analyses with comprehensive data on comorbidities which are not collected with routine outcome surveillance systems. Separate reporting of treatment outcome in pulmonary and extra-pulmonary tuberculosis should be considered.

## Competing interests

The authors declare that they have no competing interests.

## Authors' contributions

TV checked the data, led the analysis and interpretation of the data, and prepared an initial draft of the manuscript. PH and JO performed the statistical analyses and contributed to drafting the manuscript. KL contributed to designing the study, analysis and interpretation of the data, and to drafting the manuscript. PR conceived of and coordinated the project, contributed to analysis and interpretation of the data, to drafting the manuscript, and critically revised the manuscript. All authors read and approved the final manuscript.

## Pre-publication history

The pre-publication history for this paper can be accessed here:

http://www.biomedcentral.com/1471-2458/10/399/prepub
